# Mitochondrial impairment and mTORC1 signalling exhaustion define NK Cell dysfunction progression in melanoma

**DOI:** 10.1007/s00262-026-04323-0

**Published:** 2026-03-17

**Authors:** Eimear Mylod, Jack Behan, Dina Baier, Fergal C. Kelleher, Clair M. Gardiner

**Affiliations:** 1 School of Biochemistry and Immunology, Trinity Biomedical Sciences Institute, Trinity College Dublin, Dublin, Ireland; 2 School of Medicine, Trinity College Dublin, Dublin, Ireland; 3 Department of Medical Oncology, St. James Hospital, Dublin, Ireland; 4 Department of Medical Oncology, Tallaght University Hospital, Dublin, Ireland

**Keywords:** NK cells, Melanoma, Immune dysfunction, Metabolism, Mitochondria, Human

## Abstract

Cutaneous melanoma is a highly metastatic cancer which had limited treatment options until the advent of immune checkpoint inhibitors (ICI); however, only around 50% of patients respond. Natural killer (NK) cells are potent cytotoxic and cytokine producing lymphocytes which present a promising avenue for immunotherapy. However, the progressive dysfunction of NK cells during cancer development represents a major barrier to effective autologous therapies. This study investigated the metabolic and functional plasticity of circulating NK cells during melanoma progression from Stage III lymph node positive and Stage IV metastatic melanoma. Stage III patient NK cells displayed reduced mitochondrial mass, fragmented morphology and dysregulated mTORC1 activity, accompanied by impaired cytotoxicity. Stage IV patients showed severe mitochondrial fragmentation, altered mTORC1 activation and markedly reduced cytokine responsiveness. Pharmacologic restoration of mTORC1 activity using MHY1485 rescued IFN-y production in Stage III but not Stage IV patient NK cells, suggesting stage-dependent differences in metabolic responsiveness. Collectively, these findings indicate progressive metabolic and functional impairment of circulating NK cells as melanoma advances and highlight a potential window for therapeutic intervention earlier in disease progression.

The online version of this article (10.1007/s00262-026-04323-0) contains supplementary material, which is available to authorized users.

## Introduction

Cutaneous melanoma is a dispersive type of cancer with a proclivity to metastasise. This makes treatment challenging [1]. Previously, there were limited effective treatment options for patients with advanced disease (Stage III and IV); however, the introduction of immune checkpoint inhibitors (ICI) has helped transform outcomes [2]. The clinical success of ICI for melanoma has confirmed the therapeutic potential of harnessing immune effector cells to overcome immunosuppression [3]. However, only around 50% of patients will respond to ICI and so there is scope to develop immunotherapies to benefit those who don’t respond.

Natural killer (NK) cells are innate lymphocytes with potent cytotoxic and cytokine producing functions [4]. Owing to their anti-tumour effects, NK cells are of great interest for immunotherapy and have several advantages over other cellular therapy sources [5]. A significant barrier to the use of NK cells for autologous therapy is the progressive dysfunction of NK cells as cancer evolves. While there are numerous reports of rapid and long-lasting NK cell dysfunction in the immunosuppressive tumour microenvironment, there is now an appreciation of the significant impact the cancer environment has on circulating NK cell metabolism and function [6–8]. Our previous work in metastatic breast cancer patients demonstrated that NK cell dysfunction is associated with disrupted mitochondrial morphology and reduced mammalian target of rapamycin complex 1 (mTORC1) activity, linking metabolic stress to impaired effector function [9].

To optimise NK cell-based therapies, it is critical to define whether circulating NK cells from cancer patients retain sufficient metabolic and functional plasticity for therapeutic use. We hypothesised that as melanoma progresses, NK cells undergo stepwise dysfunction in which metabolic impairments progressively limit cytokine responsiveness, culminating in diminished effector function. Understanding the stage-specific functional capacity of NK cells will identify actionable windows for NK cell-based immunotherapy and inform strategies for metabolic reprogramming in melanoma patients.

Here, we observed progressive metabolic and functional impairments of peripheral blood NK cells from lymph node positive (LN+) Stage III to metastatic (Met) Stage IV melanoma patients. Stage III LN+ patients exhibited reduced mitochondrial mass, increased ROS, fragmented mitochondrial morphology, elevated mTORC1 activity at baseline and an inability to induce mTORC1 signalling following stimulation, accompanied by reduced cytotoxicity as compared to healthy donor (HD) cells. Stage IV Met patients exhibited more pronounced dysfunction, including severely fragmented mitochondria, sustained basal mTORC1 activation and a complete loss of cytokine responsiveness and effector function. Pharmacologic activation of mTORC1 using MHY1485 restored signalling and IFN-y production in LN+ , but not Met melanoma patients, highlighting stage-associated differences in metabolic and functional plasticity.

## Methods

### Patients and healthy controls

The work was performed in accordance with the Code of Ethics of the World Medical Association (Declaration of Helsinki) for experiments involving humans. Patients provided informed consent for sample and data acquisition, and the study received full ethical approval from the St. James’s Hospital Ethics Review Board. Patient samples were pseudonymised to protect the privacy rights of the patients. This study received ethical approval from the Faculty of STEM research ethics committee at Trinity College Dublin for the provision of healthy donor blood. Blood samples were obtained from healthy donors or patients with lymph node positive (Stage III) or metastatic (Stage IV) melanoma prior to commencement of treatment. A total of 48 melanoma patients were enrolled in the study from 2023 to 2025, 29 patients had Stage III LN+ disease, while 19 had Stage IV metastatic disease. Demographics and characteristics are reported in Table 1.

**Table 1:** Patient demographics

Age (years) (range)	67.1 (26—87)
Sex (M:F)	26:22

Stage (no. pt)	
LN+ stage III	29
Metastatic stage IV	19

Treatment (no. pt)	
Pembrolizumab	27
Nivolumab	8
Nivolumab/ipilimumab	9
BRAF/MEK inhibitor	1
No immunotherapy	3

Response of metastatic patients (no. pt)	
Non responder	7
Partial response	1
Complete response	10
Response unknown	1

Mutational status (no. pt)	
BRAFV600E	12
BRAFV600K	1
NRAS Codon 61	12
NRAS Codon 12	1
NRAS Codon 146	1
No mutations	9
Unknown	12

Staging category for stage III patients (AJCC 8th Edition)	
IIIA	3
IIIB	7
IIIC	15
IIID	1
Unknown	3

TILS	
Brisk	3
Non-brisk	13
Absent	2
Unknown	30

### Cell culture

Peripheral blood mononuclear cells (PBMC) were isolated by density gradient centrifugation. For confocal microscopy analysis, NK cells were identified as CD56^+^ (PeVio770; Miltenyi Biotec) and CD3^−^ (Pacific Blue; Biolegend) cells and sorted using the BD FACSAria cell sorter (BD). PBMC were cultured in RPMI 1640 (Gibco) supplemented with 10% foetal bovine serum (FBS) and 1% penicillin/streptomycin (both Gibco). Cells were stimulated for 18 h with 30 ng/ml IL-12 (Miltenyi Biotec) and 100 ng/ml IL-15 (Miltenyi Biotec). Cells were treated with brefeldin A for the final 4 h of stimulation. For the analysis of degranulation, CD107a PE (Biolegend) was added for the final 3 h of incubation. For experiments using mTORC1 activator MH1485 (MedChem Express), cells were treated for 4 h with 10 µM MH1485, with and without subsequent stimulation with 30 ng/ml IL-12 (Miltenyi Biotec) and 100 ng/ml IL-15 (Miltenyi Biotec).

### Flow cytometry analysis

A viability dye was included in every panel (LIVE/DEAD Near-IR, Bio Sciences). Cells were stained for 15 min at 4 °C with saturating concentrations of CD56 PeCy7 (HCD56, Biolegend) or PeVio770 (AF12-7H3, Miltenyi Biotec), CD3 Pacific Blue (UCHT1, Biolegend), CD69 BV785 (FN50, Biolegend), CD71 PeCy5 (CY1G4, Biolegend), CD98 PE (UM7F8, BD), CD25 APC (BC96, Biolegend) and TRAIL APC (RIK-2, Biolegend). Cells were fixed with BD cytofix for 20 min at 4 °C and subsequently permeabilised with BD perm/wash. Cells were stained at 4 °C with saturating concentrations of IFN-y AF700 (4S.B3, Biolegend), TNF-*α* BV650 (MAb11, Biolegend), ATP5b AF488 (3D5, Abcam), Perforin PerCpCy5 (dG9, Biolegend), Granzyme B BV510 (GB11, BD) and S6 ribosomal protein phosphorylated on serine 235/6 (pS6) PE (D68F8, Cell Signalling Technology) or AF488 (N7-548, BD). For analysis of cMyc FITC (SH1-26E7.1.3, Miltenyi Biotec) expression, cells were fixed and permeabilised using the FoxP3 transcription factor staining kit (eBioscience) as per manufacturer’s instructions. Samples were acquired on the BD LSR Fortessa. Full gating strategy is presented in Supplemental Fig. 1.

### Mitochondrial analysis by flow cytometry

Cells were co-stained for 30 min at 37 °C with 100 nM of Mitotracker green (Invitrogen) to measure mitochondrial mass and 100 nM of tetramethylrhodamine methyl ester (TMRM, Invitrogen) to measure mitochondrial membrane potential (MMP). Oligomycin (2 µM) and carbonyl cyanide p-trifluoro-methoxyphenyl hydrazone (FCCP, 2 µM) were used as positive and negative controls, respectively. Mitochondrial superoxide levels were measured via staining of cells for 15 min at 37 °C with MitoSOX (Thermo Fisher). Rotenone (20 µM) and MitoTEMPO (2.5 µM) were used as positive and negative controls, respectively. Cells were subsequently stained with CD56 PeCy7 and CD3 Pacific blue to identify NK cells.

### Confocal imaging

NK cells were isolated by FACS from PBMC of 3 HD, 3 LN+ and 3 Met melanoma patients. Purified NK cells were stained with 250 nM of Mitospy CMX Ros (Biolegend) for 30 min at 37 °C. Cells were washed with PBS and fixed in 2% paraformaldehyde (Sigma) for 15 min and room temperature. Following washing, cells were stained with 4′,6-diamidino-2-phenylindole dihydrochloride (DAPI, Thermo Fischer, 1/10000) for 10 min at room temperature. Cells were mounted using Mowiol (Sigma) and left to set overnight at room temperature. Cells were imaged on a Leica SP8 inverted motorised microscope equipped with a × 63/1.4 N.A. oil objective.

### Mitochondrial morphology analysis

Mitochondrial fluorescence was quantified in Fiji (http://fiji.sc/Fiji) by delineating the cells as regions of interest (ROI) and measuring the integrated density of Mitospy CMX Ros. Automated mitochondrial morphology was analysed using macros detailed in Cervantes-Silva et al. [10].

### Statistics

GraphPad Prism V10 was used for statistical analysis. A two-sided Mann–Whitney test was used to compare between two groups. A two-sided Kruskal–Wallis tests were used to compare between three or more groups with post-hoc Dunn’s test. A two-sided one sample Wilcoxon test was used to compare normalised data. For analyses of stimulation or treatment-induced responses, MFI values were normalised to the corresponding unstimulated or untreated condition for each donor to control for inter-individual and inter-experimental variability inherent to primary human samples. *P* < 0.05 was considered statistically significant.

## Results

**Fig. 1:**
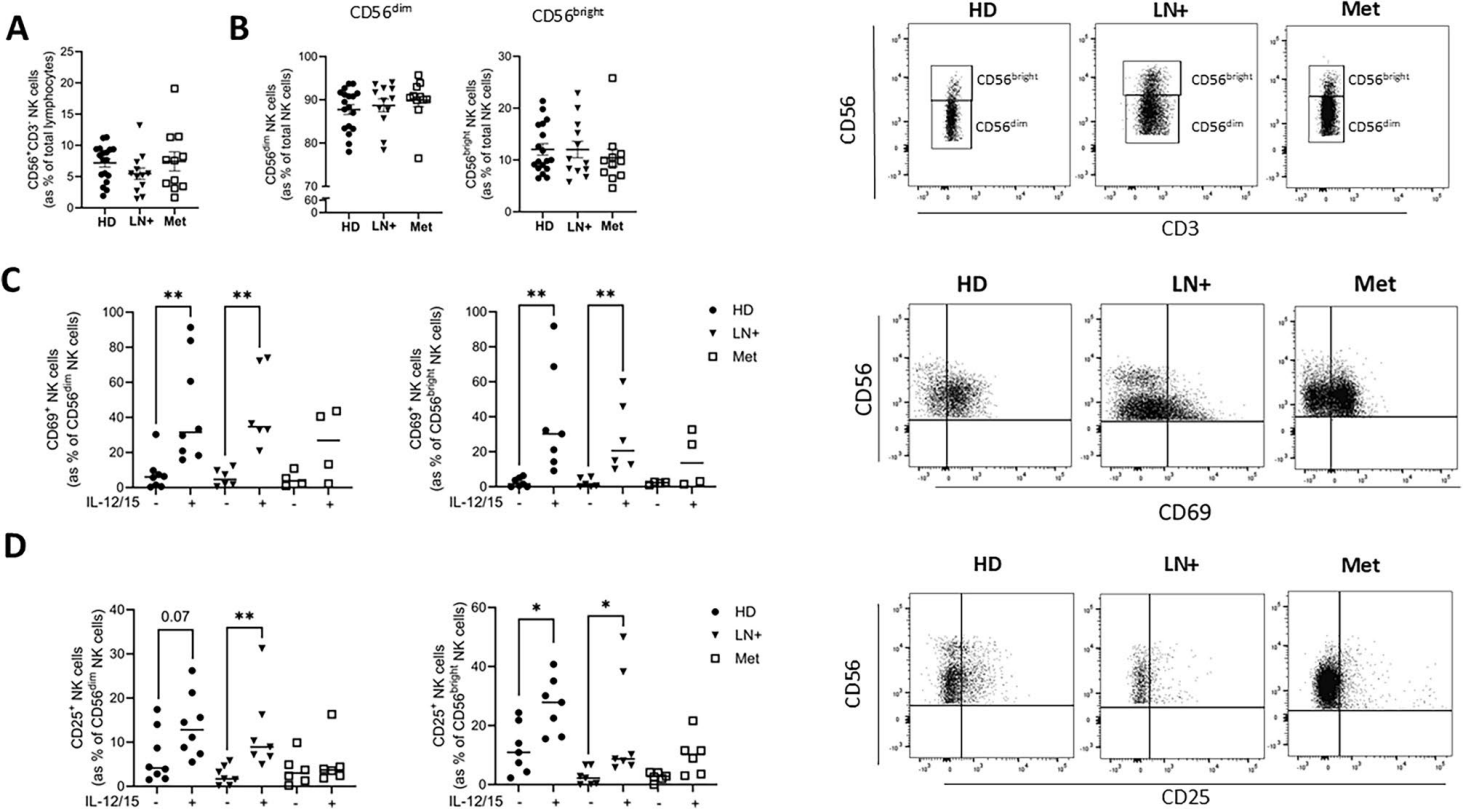
**CD25 is not increased following stimulation in Met melanoma patient NK cells.** PBMC from HD, LN+ and Met melanoma patients were isolated and stained ex vivo or following stimulation with IL-12/15. **A, B** PBMC were stained ex vivo for CD56 and CD3 and analysed by flow cytometry to identify the frequency of (A) total CD56^+^CD3^−^ NK cells and (B) CD56^dim^ and CD56^bright^ subsets. Representative dot plot showing CD56^dim^ and CD56^bright^ subsets in HD, LN+ and Met melanoma patient NK cells **C, D**. (Left) PBMC were stimulated with IL-12/15 and the frequency of (C) CD69^+^ and (D) CD25^+^ CD56^dim^ and CD56^bright^ NK cells was analysed. (Right) Representative dot plot showing (C) CD69^+^ and (D) CD25^+^ NK cells in HD, LN+ and Met melanoma patients. *p < 0.05 by Kruskal–Wallis test with post-hoc Dunn’s test

Total NK cell and subset (CD56^dim^ and CD56^bright^) frequencies in PBMC were similar between healthy donor (HD), LN+ and Met melanoma patients (Fig. 1A, B). Cytokines IL-12 and IL-15 are commonly used to activate NK cells and following stimulation, HD and LN+ melanoma patients significantly increase expression of the activation marker CD69, while met melanoma patients do not (Fig. 1C). In parallel, HD and LN+ patients significantly increased CD25, the high affinity IL-2 receptor subunit, in response to IL-12/15 stimulation, while Met melanoma patients did not (Fig. 1D). CD25 was substantially increased on CD56^dim^ HD NK cells; however, this did not reach significance (*P* = 0.07) (Fig. 1D). NK cell frequencies were comparable; however, while HD and LN+ melanoma patients induced CD69 and CD25 expression following stimulation with IL-12/15, similar responses were not seen in met melanoma patients, indicating impaired cytokine-driven activation with disease progression.

### Diminished mitochondrial mass and elevated membrane potential in NK cells from LN+ and Met melanoma patients

**Fig. 2:**
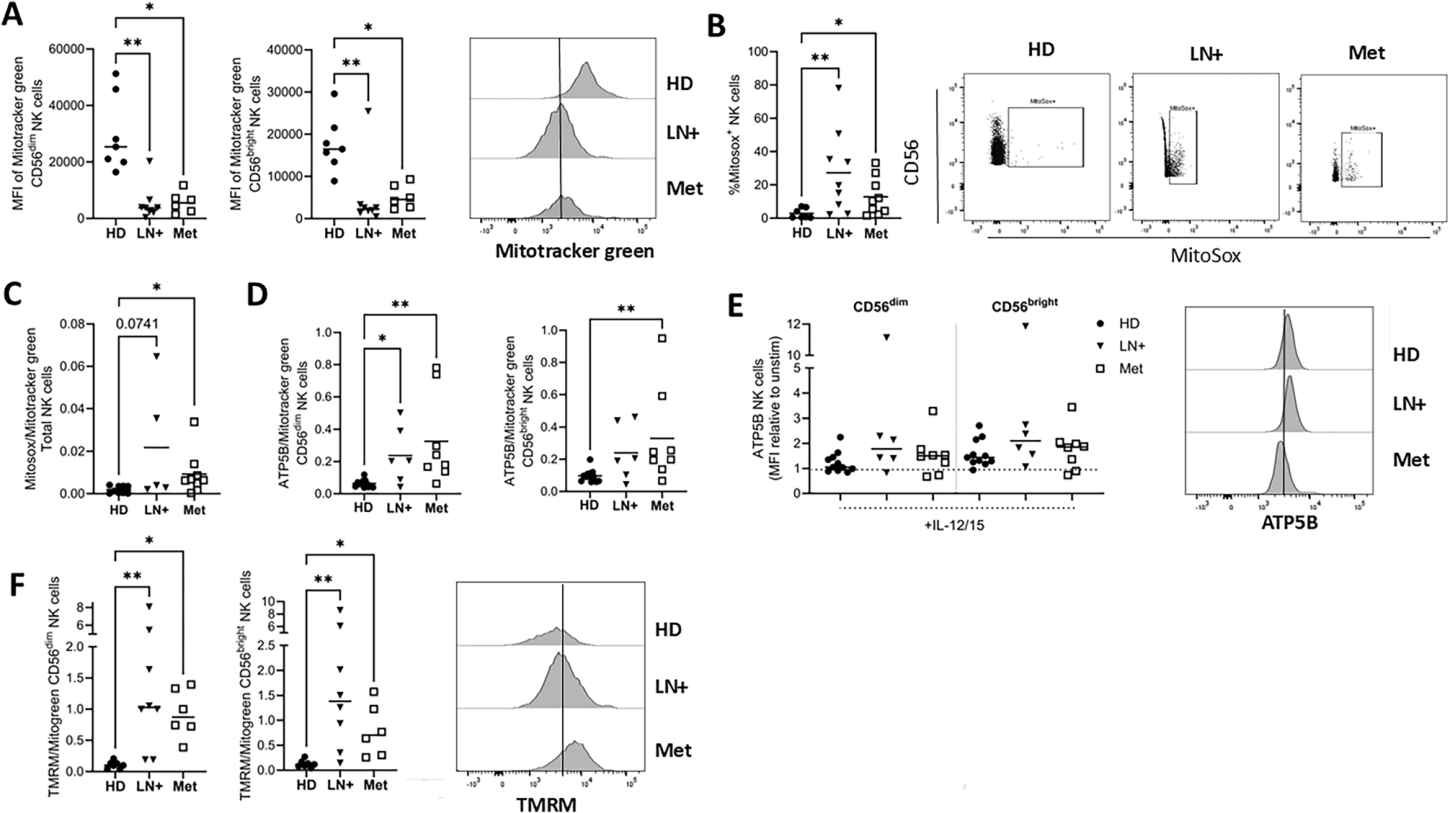
**Mitochondria from LN+ and Met melanoma patient NK cells have diminished mass and elevated membrane potential.** PBMC from HD, LN+ and Met melanoma patients were analysed ex vivo or following stimulation with IL-12/15 (ATP5B only). **A** CD56^dim^ (left) and CD56^bright^ (right) NK cells were stained ex vivo with Mitotracker green to measure mitochondrial mass. Representative histograms of HD, LN+ and Met melanoma patient NK cells stained with Mitotracker Green. **B** Total NK cells were stained ex vivo with MitoSox to measure mitochondrial ROS. Representative dot plot showing MitoSox^+^ NK cells in HD, LN+ and Met melanoma patients. **C** MFI of MitoSox normalised to average Mitotracker green per group was used to measure mitochondrial ROS per mitochondria ex vivo. **D** MFI of ATP5B normalised to average Mitotracker green per group was used to measure ATP5B levels per mitochondria of CD56^dim^ (left) and CD56^bright^ (right) NK cells ex vivo. **E** MFI of ATP5B on CD56^dim^ and CD56^bright^ NK cells following stimulation with IL-12/15 represented as a fold change relative to unstimulated cells. Representative histograms of HD, LN+ and Met melanoma patient NK cells stained with ATP5B following stimulation with IL-12/15. **F** CD56^dim^ (left) and CD56^bright^ (right) NK cells were stained ex vivo with TMRM which was normalised to Mitotracker green to measure mitochondrial membrane potential per mitochondria. Representative histograms of HD, LN+ and Met melanoma patient NK cells stained with TMRM. *p < 0.05, **p < 0.01 by Kruskal–Wallis test with post-hoc Dunn’s test or one sample Wilcoxon test as appropriate

Mitochondria are key organelles in generating biosynthetic precursors, ATP and regulating redox homeostasis in cells. Mitotracker green was used to measure total mitochondrial abundance by flow cytometry. NK cells from LN+ and Met melanoma patients had significantly lower mitochondrial mass compared to HD NK cells (Fig. 2A). Using MitoSox to measure mitochondrial ROS, LN+ and Met melanoma patients had significantly increased frequencies of MitoSox^+^ NK cells compared to HD (Fig. 2B). MitoSox fluorescence was normalised to the mean Mitotracker Green signal of the corresponding group (HD, LN+, or Met) to account for differences in mitochondrial mass. Although only a subset of NK cells were MitoSox^+^, normalisation was applied at the population level. Of note, patients had elevated mitochondrial ROS when normalised to mitochondrial mass (Fig. 2C). We also measured expression of ATP5B, a key subunit of ATP synthase complex (complex V of electron transport chain (ETC)). When normalised to mitochondrial mass, LN+ and Met melanoma patient NK cells had elevated ATP5B expression compared to HD (Fig. 2D). However, any differences are lost when NK cells are stimulated (Fig. 2E). Mitochondrial membrane potential (MMP) is required to drive ATP synthase. Using TMRM dye, LN+ and Met melanoma patients had significantly elevated MMP when normalised to mitochondrial mass (Fig. 2F). Together these results suggest there is a functional dysregulation of the ETC in NK cells from melanoma patients which is characterised by higher MMP and higher mitochondrial ROS. These results indicated that melanoma patients exhibited reduced mitochondrial mass but disproportionately increased mitochondrial ROS, ATP5B expression and membrane potential when normalised to mass, indicating ETC dysregulation.

### Altered mitochondrial morphology in LN+ and Met melanoma patient NK cells

**Fig. 3:**
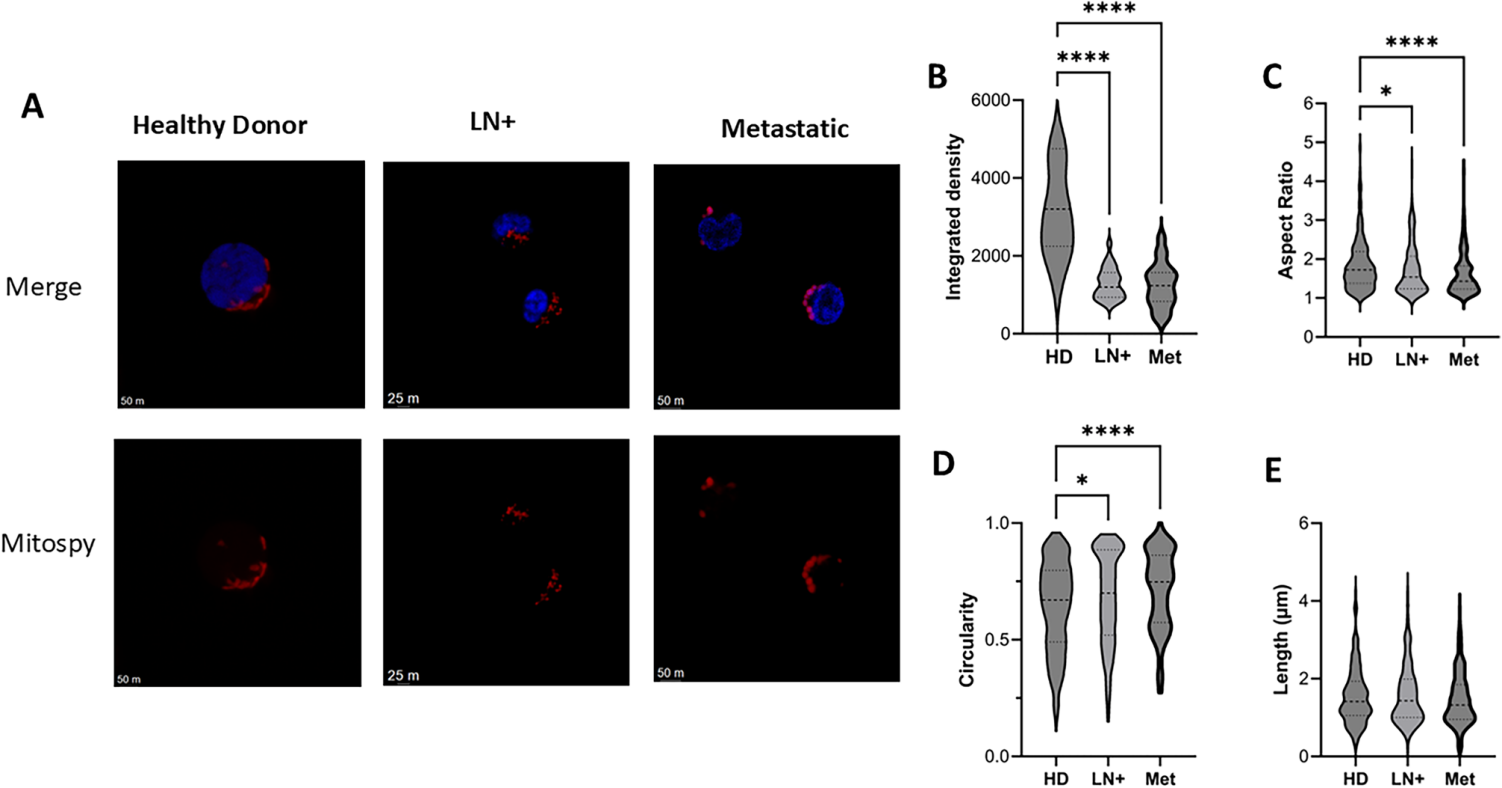
**NK cells from melanoma patients have altered mitochondrial structures.** NK cells were isolated from PBMC of 3 HD, 3 LN+ and 3 Met melanoma patients and stained with MitoSpy CMXRos and DAPI. **A** Representative confocal images of DAPI (blue) and MitoSpy CMXRos (red) stained NK cells. **B** Integrated density measurement of MitoSpy CMXRos in NK cells from HD (*n* = 44 individual NK cells), LN+ (*n* = 27 individual NK cells) and Met (*n* = 35 individual NK cells) melanoma patients. Mitochondrial morphology analysis was completed using macros from Cervantes-Silva et al*.* HD; *n* = 364 mitochondria, LN+ ; *n* = 193 mitochondria, Met; *n* = 185 mitochondria. **C** Aspect ratio of mitochondria (length/width), 1 is a circle, > 1 is indicative of an elongated mitochondria **D** Circularity of mitochondria (the ratio between a perimeter of a perfect circle of the same area and the cell perimeter), 1 is a circle, 0 is an elongated mitochondria. **E** Length of mitochondria in µm. *p < 0.05, ****p < 0.001 by Kruskal–Wallis test with post-hoc Dunn’s test

Mitochondrial mass measures total mitochondrial abundance. However, mitochondrial structures are dynamically regulated, and their architecture fluctuates to reflect functions [11]. In particular, fused or elongated mitochondria are associated with healthy, active mitochondria that are efficiently organised for oxidative phosphorylation (Oxphos). In contrast, fragmented mitochondria are associated with dysregulated metabolic outputs and mitophagy. Based on our data above, we hypothesised that the mitochondria in patient NK cells would have altered mitochondrial organisation which would contribute to their altered mitochondrial outputs. Indeed, confocal microscopy revealed pronounced differences in mitochondrial structures between the patient cohorts. HD NK cells had a more elongated mitochondrial morphology, as indicated by the greater aspect ratio (length/width) and lower circularity (1 is a perfect circle indicative of a fragmented phenotype) values (Fig. 3A, C, D). LN+ melanoma patients had values indicative of a circular, less networked mitochondria, with this phenotype even more pronounced in the Met melanoma patients (Fig. 3A, C, D). Notably, in both LN+ and Met melanoma patients, the integrated density of MitoSpy for individual NK cells was significantly lower than HD (Fig. 3B). This suggests a lower mitochondrial mass in these patients, which supports our flow cytometric data. Thus, there is a progressive metabolic dysfunction of mitochondrial organisation in circulating NK cells from patients as disease progresses and this is likely responsible for aberrant ETC outputs. Overall, confocal imaging showed that NK cells from LN+ and metastatic melanoma patients exhibit a shift from elongated, fused networks to fragmented, circular mitochondria, consistent with progressive mitochondrial organisational dysfunction.

### Induction of transferrin receptor is reduced in NK cells from Met melanoma patients

**Fig. 4:**
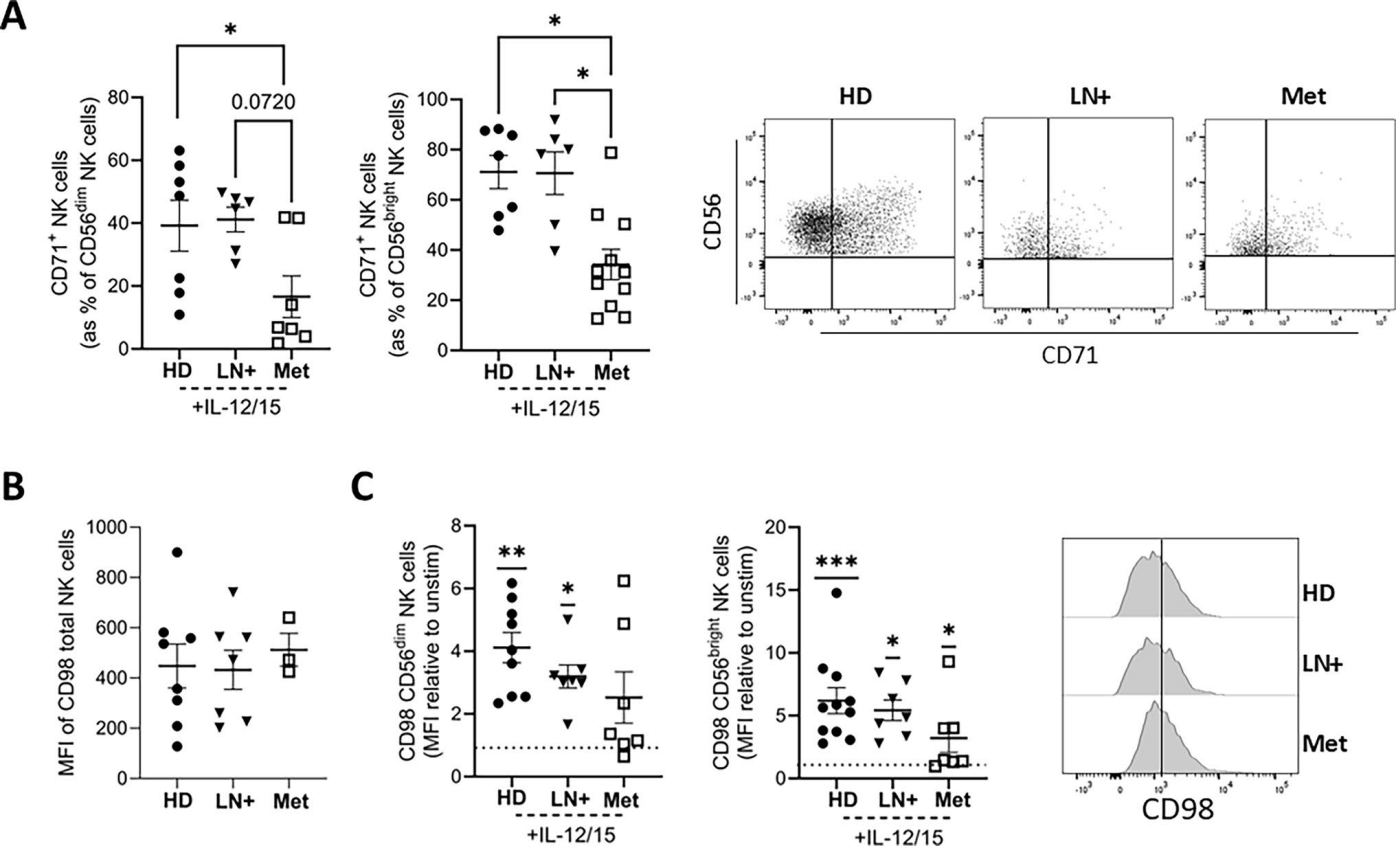
**Induction of transferrin receptor is reduced in Met patients.** PBMC were isolated from HD, LN+ and Met melanoma patients and analysed directly ex vivo or following stimulation with IL-12/15. **A** CD71^+^ CD56^dim^ (left) and CD56^bright^ (right) NK cells following stimulation with IL-12/15. Representative dot plot showing CD71^+^ NK cells in HD, LN+ and Met patients. **B** MFI of CD98 on total NK cells ex vivo*.* **C** MFI of CD98 on CD56^dim^ (left) and CD56^bright^ (right) NK cells following stimulation with IL-12/15 represented as a fold change relative to unstimulated cells. Representative histograms of HD, LN+ and Met patient NK cells stained with CD98 following stimulation with IL-12/15. *p < 0.05, **p < 0.01, ***p < 0.001 by Kruskal–Wallis test with post-hoc Dunn’s test or one sample Wilcoxon test as appropriate

The presence of defects in NK cell mitochondria indicated disruptions in NK cell metabolism. The transport of nutrients such as iron or amino acids into the cell is also essential for supporting increased metabolic rates following stimulation. For example, iron (Fe) is a key component of ETC complexes I-IV where it functions to transfer electrons, culminating in ATP production. In human NK cells, the transferrin receptor, CD71, is normally significantly upregulated upon cytokine stimulation; however, its induction was significantly lower on the surface of CD56^dim^ and CD56^bright^ NK cells from Met melanoma patients, compared to HD and LN+ melanoma NK cells (Fig. 4A). Directly ex vivo*,* there were no significant differences in CD98 expression, a protein which regulates amino acid transport, on total NK cells (Fig. 4B). Furthermore, stimulation with IL-12/15 causes comparable increases in expression of CD98 (Fig. 4C). In summary, while basal and cytokine-induced expression of CD98 was preserved, NK cells from Met melanoma patients failed to appropriately upregulate the iron transporter CD71 after IL-12/15 stimulation. These findings highlight dysregulation of specific nutrient receptors on immune cells during melanoma progression and advancing metabolic dysregulation with worsening disease.

### Functional defects in LN+ and Met melanoma patient NK cells

**Fig. 5:**
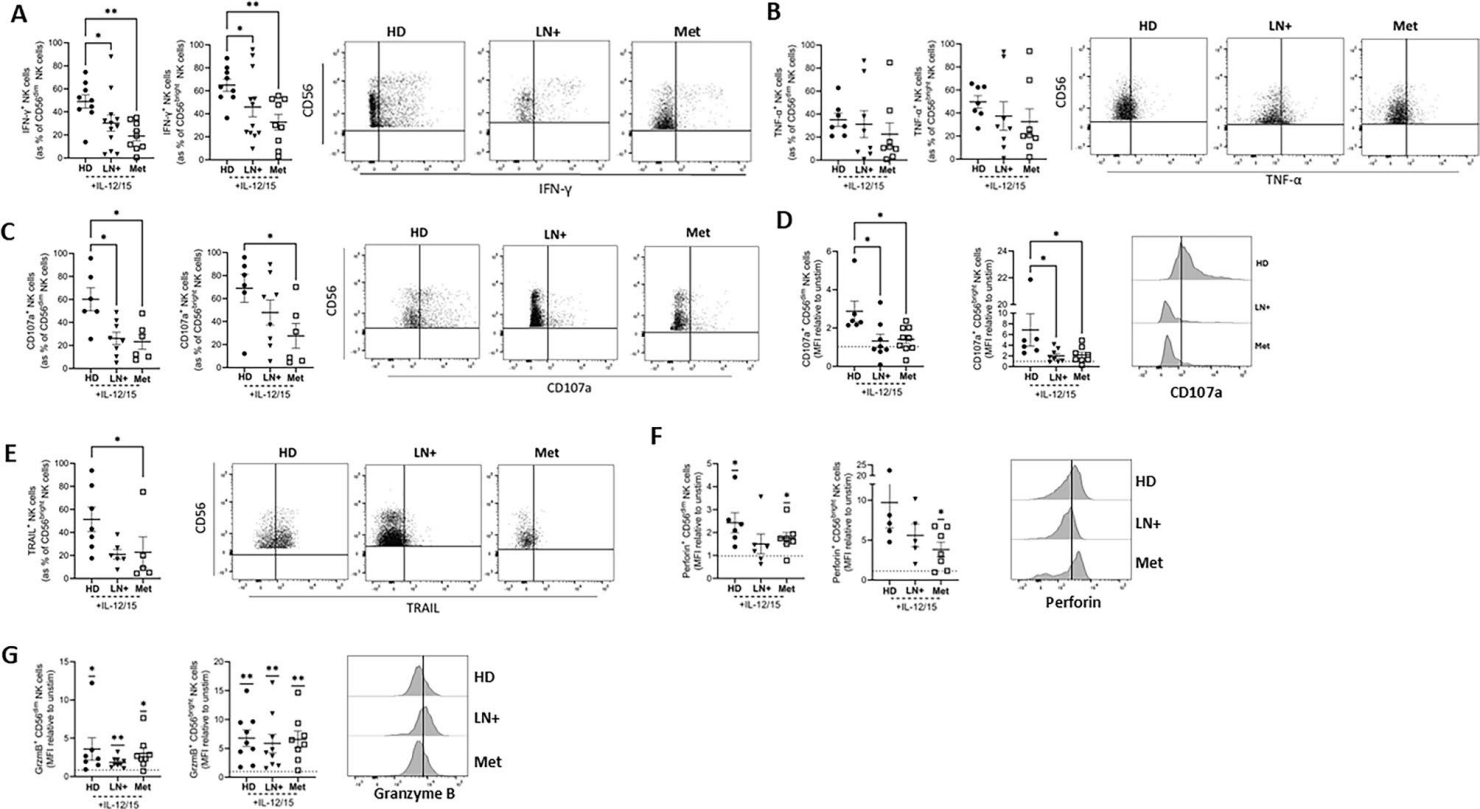
**NK cells from melanoma patients have severe functional defects.** PBMC from HD or LN+ or Met melanoma patients were analysed following stimulation with IL-12/15. **A** Frequency of IFN-γ^+^ CD56^dim^ (left) and CD56^bright^ (right) NK cells following stimulation. Representative dot plot showing IFN-γ^+^ NK cells in HD, LN+ and Met patients. **B** Frequency of TNF-*α*^+^ CD56^dim^ (left) and CD56^bright^ (right) NK cells following stimulation. Representative dot plot showing TNF-*α*^+^ NK cells from HD, LN+ and Met melanoma patients. **C** Frequency of CD107a^+^ CD56^dim^ (left) and CD56^bright^ (right) NK cells following IL-12/15 stimulation. Representative dot plot showing CD107a^+^ NK cells from HD, LN+ and Met melanoma patients. **D** MFI of CD107a represented as a fold change relative to unstimulated cells. Representative plot showing MFI of CD107a^+^ NK cells from HD, LN+ and Met melanoma patients. **E** Frequency of TRAIL^+^ CD56^bright^ NK cells following stimulation. Representative dot plot showing TRAIL^+^ NK cells from HD, LN+ and Met melanoma patients. **F** MFI of perforin expressing NK cells following stimulation represented as a fold change relative to unstimulated cells. Representative plot showing MFI of perforin^+^ NK cells from HD, LN+ and Met melanoma patients. **G** MFI of Granzyme B expressing NK cells following stimulation represented as a fold change relative to unstimulated cells. Representative plot showing MFI of Granzyme B^+^ NK cells from HD, LN+ and Met melanoma patients. *p < 0.05, **p < 0.01 by Kruskal–Wallis test with post-hoc Dunn’s test or one sample Wilcoxon test as appropriate

As NK cell metabolism is central to driving increased NK cell function following stimulation, we explored the capacity of NK cells to engage important effector functions when activated. Production of IFN-γ by LN+ and Met melanoma patient NK cells was significantly reduced following stimulation compared to HD (Fig. 5A), while TNF-*α* trended lower in the patients; however, this was not statistically significant (Fig. 5B). For both LN+ and Met melanoma patients, there was a clear deficit in NK cell cytotoxicity, with the frequency (Fig. 5C) and MFI (Fig. 5D) of degranulation marker CD107a significantly decreased for both groups. Induction of the death receptor TRAIL, which is preferentially expressed on CD56^bright^ NK cells and normally increases following cytokine stimulation, was reduced relative to HD, reaching statistical significance in NK cells from Met melanoma patients (Fig. 5E). However, not all elements of NK cell cytotoxicity were impacted as expression of perforin and granzyme B increased normally following stimulation (Fig. 5F, G). Taken together, NK cells from melanoma patients exhibited markedly reduced IFN-γ production, impaired degranulation despite preserved induction of perforin and granzyme B, indicating selective and progressively severe defects in NK cell effector function.

**Fig. 6:**
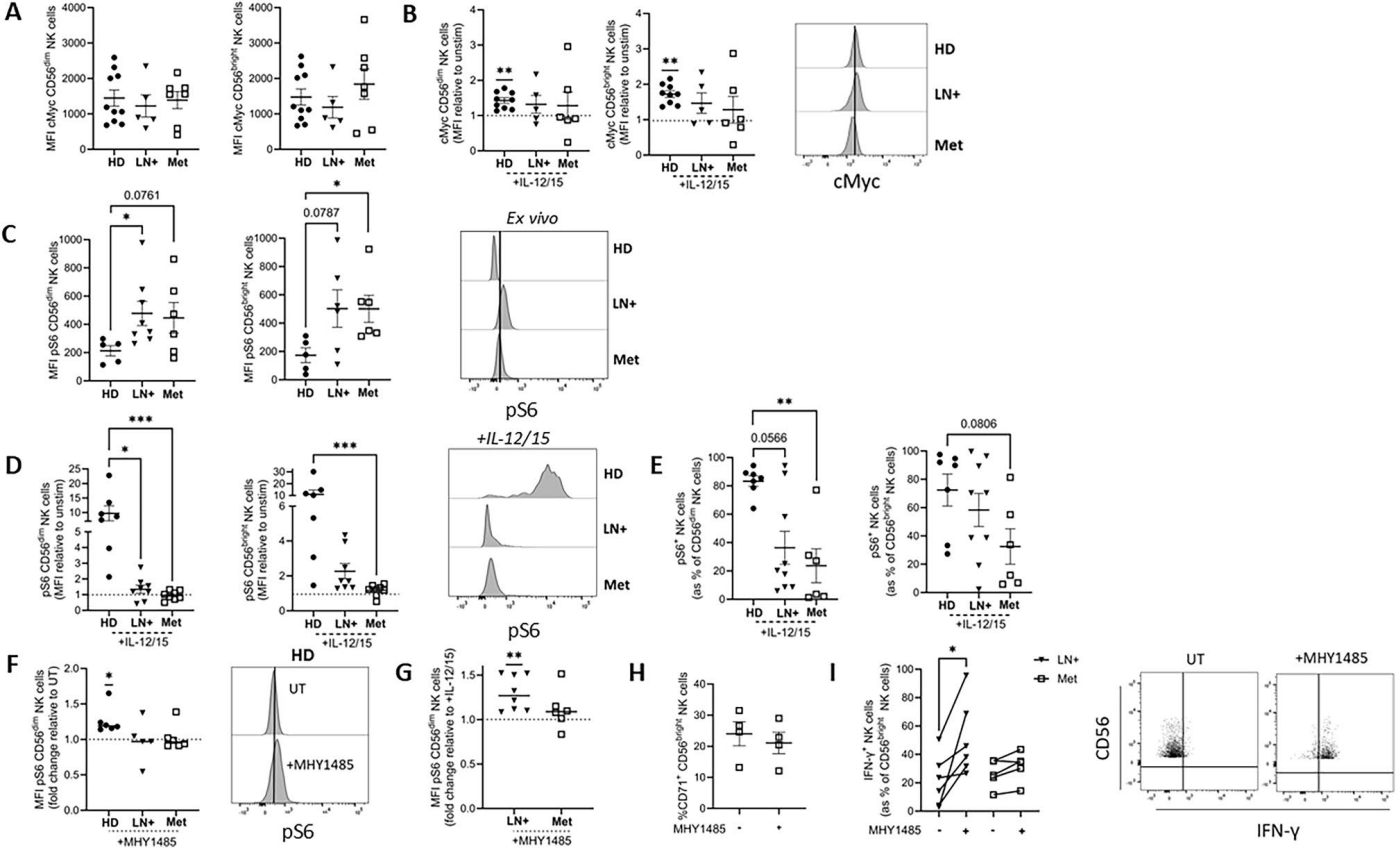
**mTORC1 signalling is defective in melanoma patient NK cells but be rescued by an mTORC1 activator in LN+ patients.** PBMC from HD, LN+ or Met melanoma patients were analysed ex vivo or following stimulation with IL-12/15. (A,B) MFI of cMyc on CD56^dim^ (left) and CD56^bright^ (right) NK cells **A** ex vivo and **B** following stimulation with IL-12/15 represented as a fold change relative to unstimulated cells. Representative histograms of HD, LN+ and Met patient NK cells stained with cMyc following stimulation with IL-12/15. (**C**) MFI of pS6^+^ CD56^dim^ (left) and CD56^bright^ (right) NK cells ex vivo. Representative plot showing pS6 MFI in HD, LN+ and Met melanoma patients. Representative histograms of HD, LN+ and Met melanoma patient NK cells stained with pS6 ex vivo. **D** MFI of pS6^+^ CD56^dim^ (left) and CD56^bright^ (right) NK cells following IL-12/15 stimulation. **E** Frequency of pS6^+^ CD56^dim^ (left) and CD56^bright^ (right) NK cells following stimulation with IL-12/15. **F** MFI pS6 CD56^dim^ NK cells following 4 h treatment with 10 µM MHY1485 represented as a fold change relative to untreated (UT) cells. Representative plot showing pS6 MFI in untreated and MHY1485 treated HD cells. **G** MFI of pS6 expressing CD56^dim^ NK cells following 4 h pre-treatment with 10 µM MHY1485 and stimulation with IL-12/15 for 18 h. Data represented as a fold change relative to IL-12/15 stimulated cells. **H** CD71^+^ CD56^bright^ NK cells from Met patients following stimulation with IL-12/15 with and without pre-treatment with 10µM MHY1485. **I** IFN-γ^+^ CD56^bright^ NK cells from LN+ and Met melanoma patients following stimulation with IL-12/15 with and without pre-treatment with 10 µM MHY1485. Representative dot plot showing IFN-y^+^ NK cells in LN+ patients. *p < 0.05, **p < 0.01, ***p < 0.01 by Kruskal–Wallis test with post-hoc Dunn’s test or one sample Wilcoxon test

It is known that both mTORC1 and cMyc play important roles in regulating NK cell metabolism, with both driving CD71 expression while mTORC1 also contributes to IFN-*γ* production [12, 13]. We first measured expression of transcription factor cMyc by flow cytometry and levels were equivalent when analysed directly ex vivo across all groups (Fig. 6A). In NK cells from healthy donors, cMyc was robustly and reproducibly upregulated after 18 h stimulation with IL-12/15. However, patients varied in terms of responses, with some patients considerably increasing cMyc expression, while others had a marked decrease (Fig. 6B).

Finally, we explored pS6, a readout of mTORC1 activity, an important metabolic regulator in NK cells. Ex vivo, pS6 was significantly elevated in both LN+ and Met melanoma patients (Fig. 6C). However, following stimulation with IL-12/15, both patient groups failed to upregulate pS6 (Fig. 6D, E). As mTORC1 was clearly dysregulated in NK cells from melanoma patients, we employed MHY1485, an mTORC1 activator, to determine if mTORC1 signalling could be rescued in patient NK cells. While in HD NK cells, treatment with MHY1485 for four hours resulted in increased pS6, similar increases were not present in LN+ or Met patients (Fig. 6F). Pre-treatment with MHY1485 prior to stimulation increased pS6 in LN+ patients (Fig. 6G). As CD71 induction was significantly reduced in Met patients and CD71 is a downstream target of mTORC1, we next investigated whether pre-treatment with MHY1485 could rescue CD71 induction. There were no significant differences in CD71 frequency on CD56^bright^ NK cells following stimulation with IL-12/15 with or without pre-treatment with MHY1485 (Fig. 6H). Finally, we sought to determine if pre-treatment with MHY1485 could enhance production of IFN-γ by LN+ and Met patient NK cells. LN+ patients had a marked increase in IFN-γ^+^ CD56^bright^ NK cells following pre-treatment with MHY1485 prior to stimulation; however, the same was not seen in NK cells from Met melanoma patients (Fig. 6I). These data indicate that NK cells from melanoma patients possess dysregulated metabolic signalling characterised by variable cMyc induction, elevated basal but blunted cytokine-induced mTORC1 activity and limited responsiveness to pharmacologic mTORC1 activation, indicating progressively irreversible metabolic dysfunction with disease advancement.

## Discussion

NK cell dysfunction is a common feature of cancer that compromises the efficacy of cellular immunotherapy. In this study, we demonstrate that NK cell metabolic dysfunction in human melanoma progresses in a stage-dependent manner, characterised by changes in mitochondrial integrity, mTORC1 signalling and effector function. Stage III LN+ patient NK cells exhibit markers of early dysfunction, while Met stage IV patients display more pronounced alterations across these parameters.

Mitochondria are central to NK cell metabolism, supporting energy production through Oxphos and are required for the synthesis of effector molecules, making the mitochondria highly informative of the metabolic status of a cell [14]. We observed marked mitochondrial abnormalities in our melanoma patient-derived NK cells, including reduced mass, hyperpolarised membranes, increased ROS and fragmented, circular mitochondria, consistent with prior reports in breast and liver cancer [9, 15]. Mitochondrial defects underpin a number of pathological conditions, including HIV, obesity and cancer, and in this instance may reflect impaired mitochondrial fusion and reduced bioenergetic flexibility [16]. The most pronounced defects are present in the Met melanoma patients, suggesting the decrease in mass proceeds changes in mitochondrial morphology. These data further underscore the relationship between mitochondrial dynamics and NK cell function and highlight how targeting mitochondrial dynamics as a potential therapeutic approach could help restore NK cell function, as reported for T cells [17–19]. Of interest, the allogeneic transfer of mitochondria improves metabolic fitness [20] and enhanced NK cell anti-tumour efficacy [21], presenting this as a potential approach to engineer therapies in melanoma patients and circumvent mitochondrial damage. CD71 and CD98 constitute key nutrient transporters that support NK cell metabolism by facilitating iron and amino acid uptake, respectively, which are essential for mitochondrial biogenesis, Oxphos and thus downstream effector functions. Impaired induction of CD71 provides a mechanistic link between defective iron uptake and the mitochondrial dysfunction observed in Met melanoma patient NK cells.

Cytokine production and cytotoxicity are the central functions of NK cells. Production of the prototypic NK cell cytokine IFN-γ was decreased in Met patients, and in almost all LN+ patients. While degranulation, as measured by CD107a, was dampened in both LN+ and Met patients, induction of perforin and granzyme B was normal in both. Defects in circulating NK cell cytotoxicity during cancer are commonly found, although the mechanisms of this dysfunction likely vary depending on cancer type, stage or pre-clinical model [22]. These data are consistent with defects in secretion or cytoskeletal dynamics, rather than protein synthesis may be responsible for the specific defect in NK cell degranulation in melanoma. Together, these data support functional dysregulation and extend our previous findings of severe metabolic dysregulation in circulating NK cells from metastatic breast cancer and neuroblastoma to another solid tumour [8, 9, 23]. The severe functional defects observed here are likely a direct consequence of the profound metabolic and mitochondrial dysregulation evident in these patients as degranulation and cytokine production are highly energy-dependent processes [23, 24].

As NK cell metabolism underpins function, which is severely impacted in melanoma patients, we next sought to examine the role of the mTORC1 protein complex, a central player in the control of NK cell metabolism and thus function [12]. Previous reports have highlighted specific defects in the mTORC1 pathway in NK cells across several pathological contexts characterised by NK cell dysfunction [9, 23, 25, 26]. Here, both LN+ and Met melanoma patient NK cells exhibited elevated basal levels of pS6 ex vivo but failed to further upregulate pS6 upon cytokine simulation, mirroring our findings in metastatic breast cancer and neuroblastoma [9, 23]. This is consistent with a state of chronic mTORC1 activation and reduced signalling responsiveness. As mTORC1 is essential in the production of IFN-γ, cytotoxicity and downstream targets like CD71, this links the lower induction of mTORC1 readouts and reduced function [12]. Overall, these data support the idea that disrupted mTORC1 signalling underpins NK cell dysfunction in cancer and presents mTORC1 as a common endpoint for functional exhaustion across a range of disease states.

While mTORC1 is consistently dysfunctional in our patient groups, cMyc induction is variable in the Met patients, with some patients having robust upregulation when stimulated, while others show a marked decrease in expression. This was accompanied by variable inductions of the amino acid transporter CD98 following stimulation in the Met melanoma group. As cMyc protein expression is sensitive to the supply of amino acid through transporters, this links these two elements of NK cell function [13, 27]. Interestingly, the patients with no induction of cMyc also had the lowest levels of degranulation and IFN-γ production, further linking these facets of NK cell function and highlighting the heterogeneity of individual patients’ ability to engage transcriptional programmes downstream of signalling. Further studies in larger patient cohorts will be required to further dissect the heterogeneous relationship between cMyc and NK cell effector function in melanoma patients.

Therapeutically targeting metabolic signalling via mTORC1 is of interest to maintain NK cell function in cancer and has been shown to enhance NK cell metabolic fitness and anti-tumour responses [27–29]. Here, we employed an mTORC1 activator MHY1485 to attempt to rescue patient mTORC1 signalling. While direct short-term MHY1485 treatment enhanced phosphorylation of S6 in healthy donor cells, it failed to induce similar increases in patient cells, potentially related to their elevated basal pS6 levels. However, pre-treatment with MHY1485 prior to stimulation enhanced pS6 significantly in NK cells from LN+ melanoma patients, accompanied by a restoration of IFN-γ production. In Met melanoma patients, there was some evidence of restoration of pS6 levels; however this was not accompanied by increased IFN-γ production, suggesting a downstream block in effector function in metastatic patient NK cells. This further underscores the pronounced functional impairment observed in Met melanoma patient NK cells. Given the short duration of MHY1485 treatment used in this study, our analyses focussed on acute metabolic signalling and effector function. Examining the impact of mTORC1 modulation on NK cell proliferation and expansion, and how metabolic reprogramming translates to NK cell anti-tumour activities in more complex settings represent an important area for future work.

While our findings highlight intrinsic metabolic and functional impairments in NK cells from LN+ and Met melanoma patients, we cannot exclude the possibility that some observed findings are influenced by interactions with other immune cells within the experimental set up. Nevertheless, these results remain highly relevant as they reflect NK cell behaviour within the complex, mixed cell environment in vivo*.*

Overall, these findings underscore the progressive nature of NK cell metabolic dysfunction in cancer, linking mitochondrial integrity, mTORC1 signalling and downstream effector function. Importantly, our data suggest that dysfunction in stage III LN+ patients is somewhat reversible, offering a potential window for therapeutic intervention through metabolic reprogramming to restore autologous NK cell function. In contrast, stage IV metastatic patients may be amenable to allogeneic NK cell-based therapies, such as CAR-NK cells, to circumvent the deeper metabolic issues affecting their cells. Tailoring immunotherapeutic strategies to disease stage may improve outcomes and overcome NK cell exhaustion in melanoma and potentially other cancers.

## Electronic supplementary material

Below is the link to the electronic supplementary material.

Supplementary material 1 (DOCX 183 kb)

## Data Availability

All data supporting the findings of this study are available within the paper.

## References

[1] Hsieh M-Y et al (2024) Melanoma biology and treatment: a review of novel regulated cell death-based approaches. Cancer Cell Int 24(1):63 38336727 10.1186/s12935-024-03220-9 PMC10858604

[2] Chang C-Y et al (2020) Immune checkpoint inhibitors and adverse events in advanced melanoma. JAMA Network Open 3(3):e201611 32211869 10.1001/jamanetworkopen.2020.1611 PMC7097702

[3] Xuan J et al (2024) Insights for the immunotherapy in malignant melanoma: a new revolution. Clini cancer bulletin 3(1):21

[4] Mace EM (2022) Human natural killer cells: form, function, and development. J allergy clini immunol 151(2):371 10.1016/j.jaci.2022.09.022 PMC9905317 36195172

[5] Ma S, Yu J, Caligiuri MA (2025) Natural killer cell–based immunotherapy for cancer. J Immunol 10.1093/jimmun/vkaf036 PMC12311385 40246292

[6] Mylod E et al (2024) Real-time ex vivo monitoring of NK cell migration toward obesity-associated oesophageal adenocarcinoma following modulation of CX3CR1. Sci Rep 14(1):4017 38369570 10.1038/s41598-024-54390-5 PMC10874956

[7] Dean I et al (2024) Rapid functional impairment of natural killer cells following tumor entry limits anti-tumor immunity. Nat Commun 15(1):683 38267402 10.1038/s41467-024-44789-z PMC10808449

[8] Zu S et al (2024) Changes in subset distribution and impaired function of circulating natural killer cells in patients with colorectal cancer. Sci Rep 14(1):12188 38806640 10.1038/s41598-024-63103-x PMC11133342

[9] Slattery K et al (2021) TGFβ drives NK cell metabolic dysfunction in human metastatic breast cancer. J Immunother Cancer 9(2):e002044 33568351 10.1136/jitc-2020-002044 PMC7878131

[10] Cervantes-Silva MP et al (2022) The circadian clock influences T cell responses to vaccination by regulating dendritic cell antigen processing. Nat Commun 13(1):7217 36470865 10.1038/s41467-022-34897-z PMC9722918

[11] Ghosh S, Dutta R, Goswami D, Ghatak D, De R, (2025) Mitochondrial dynamics and metabolic attributes regulate function of natural killer cell and infiltration in tumor microenvironment modulating disease progression. Biochimica Biophys Acta Rev Cancer 10.1016/j.bbcan.2025.189471 41075850

[12] Donnelly RP et al (2014) mTORC1-dependent metabolic reprogramming is a prerequisite for natural killer cell effector function. J immunol. 193(9):4477 (**(Baltimore, Md. : 1950)**) 25261477 10.4049/jimmunol.1401558 PMC4201970

[13] Loftus RM et al (2018) Amino acid-dependent cMyc expression is essential for NK cell metabolic and functional responses in mice. Nat Commun 9(1):2341 29904050 10.1038/s41467-018-04719-2 PMC6002377

[14] Gardiner CM (2019) NK cell metabolism. J Leukocyte Biol 105(6):1235 30676653 10.1002/JLB.MR0718-260R

[15] Zheng X et al (2019) Mitochondrial fragmentation limits NK cell-based tumor immunosurveillance. Nat Immunol 20(12):1656–1667 31636463 10.1038/s41590-019-0511-1

[16] Verhezen T et al (2025) Powering immunity: mitochondrial dynamics in natural killer cells. Trends Mol Med. 10.1016/j.molmed.2025.04.004 40393875

[17] Buck MD et al (2016) Mitochondrial dynamics controls T cell fate through metabolic programming. Cell 166(1):63–76 27293185 10.1016/j.cell.2016.05.035 PMC4974356

[18] Baldwin JG et al (2024) Intercellular nanotube-mediated mitochondrial transfer enhances T cell metabolic fitness and antitumor efficacy. Cell 187(23):6614-6630 e21 39276774 10.1016/j.cell.2024.08.029 PMC11623344

[19] Surace L et al (2021) Polarized mitochondria as guardians of NK cell fitness. Blood Adv. 5(1):26 33570622 10.1182/bloodadvances.2020003458 PMC7805327

[20] Harada S et al (2022) Intercellular mitochondrial transfer enhances metabolic fitness and anti-tumor effects of CAR t cells. Blood. 140 (Supplement 1).

[21] Kim S-H et al (2023) Enhancement of the anticancer ability of natural killer cells through allogeneic mitochondrial transfer. Cancers 15 12 10.3390/cancers15123225 PMC10296914 37370835

[22] Cong J et al (2018) Dysfunction of natural killer cells by FBP1-induced inhibition of glycolysis during lung cancer progression. Cell Metab 28(2):243-255 e5 30033198 10.1016/j.cmet.2018.06.021

[23] Slattery K et al (2022) Frontiers | Heightened metabolic responses in NK cells from patients with neuroblastoma suggests increased potential for immunotherapy. Frontiers Oncol. 12. 10.3389/fonc.2022.1004871 PMC9585418 36276144

[24] Keating SE et al (2016) Metabolic reprogramming supports IFN-gamma production by CD56bright NK cells. J Immunol 196(6):2552–2560 26873994 10.4049/jimmunol.1501783

[25] Tobin LM, Mavinkurve M, Carolan E, Kinlen D, O'Brien EC, Little MA, Finlay DK, Cody D, Hogan AE, O'Shea D (2017) NK cells in childhood obesity are activated, metabolically stressed, and functionally deficient. JCI Insight 10.1172/jci.insight.94939 PMC5752310 29263296

[26] Michelet X, Dyck L, Hogan A et al (2018) Metabolic reprogramming of natural killer cells in obesity limits antitumor responses. Nat Immunol. 10.1038/s41590-018-0251-7 30420624 10.1038/s41590-018-0251-7

[27] Choi C, Finlay DK (2021) Optimising NK cell metabolism to increase the efficacy of cancer immunotherapy. Stem Cell Res Ther 12(1):320 34090499 10.1186/s13287-021-02377-8 PMC8180160

[28] Zhu H et al (2020) Metabolic reprograming via deletion of CISH in human iPSC-derived NK cells promotes in vivo persistence and enhances anti-tumor activity-PubMed. Cell stem cell. 27 2 10.1016/j.stem.2020.05.008 PMC7415618 32531207

[29] Delconte R et al (2016) CIS is a potent checkpoint in NK cell-mediated tumor immunity - PubMed. Nat immunol 17 7 10.1038/ni.3470 27213690

