# Supplementary material for: Mitochondrial impairment and mTORC1 signalling exhaustion define NK Cell dysfunction progression in melanoma
Source: Cancer Immunol Immunother. 2026 Mar 17;75(4):111. doi: 10.1007/s00262-026-04323-0 (PMC12996465; doi:10.1007/s00262-026-04323-0)
Supplement: Supplementary file 1 — Supplementary material 1 (DOCX 183 kb) [file 262_2026_4323_MOESM1_ESM.docx]

*
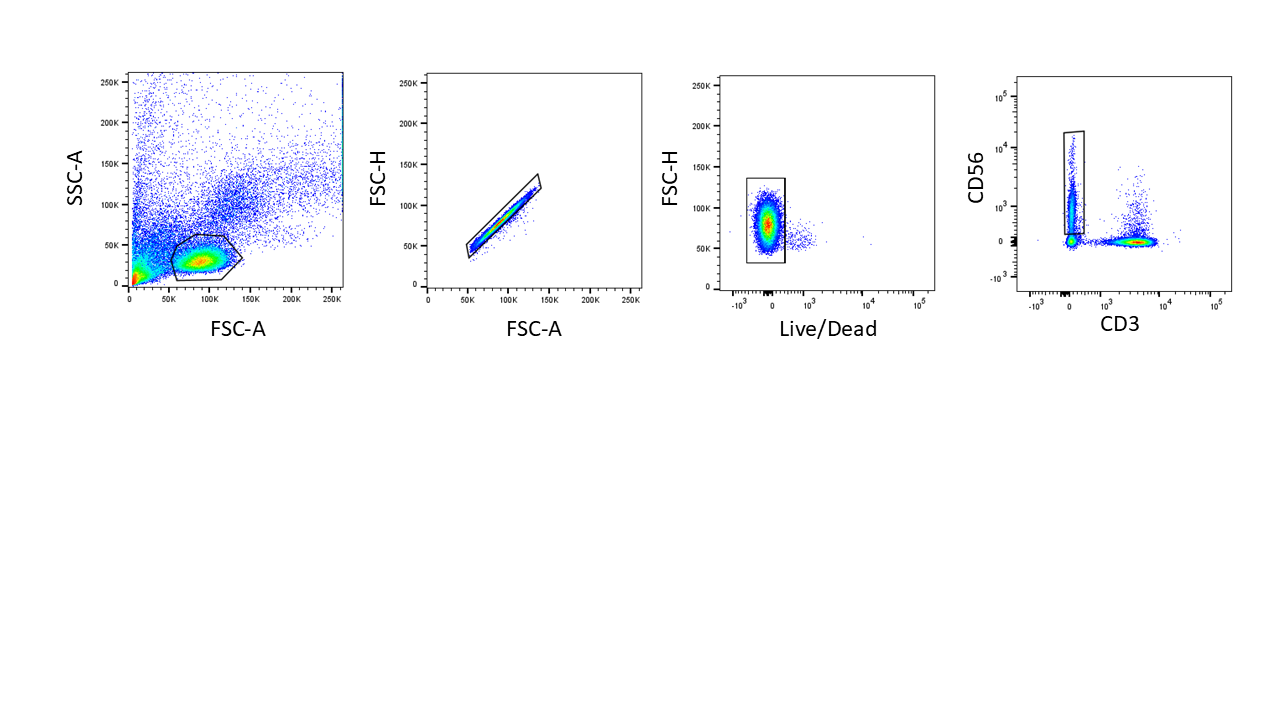
*

Supplemental Figure 1: Representative gating strategy used to define NK cells. The lymphocyte population was gated on using the SSC-A and FSC-A. Following this to remove doublets single cells were gated on within FSC-H and FSC-A. FSC-H and Live/Dead staining were used to gate on live cells. Finally, NK cells were gated as CD56^+^CD3^-^ cells within this gate.
